# Polarization and rule of law crisis—insights from Poland

**DOI:** 10.1007/s12286-025-00630-5

**Published:** 2025-04-02

**Authors:** Magdalena Solska

**Affiliations:** University of Fribourg, Fribourg, Switzerland

**Keywords:** Political polarization, Rule of law, Crisis, Poland, Strategy

## Abstract

Most scholarly arguments related to the judicial reform in Poland highlight intentional demolition of democratic standards by the PiS-led government and the subordination of the judiciary to the executive. This article puts forward a theoretical argument that factors polarizing strategy of incumbents and opposition into the analysis of the rule of law crisis. Whereas PiS-led government is responsible for devastation of the judiciary undermining the rule of law in the country, the new government’s continued polarizing strategy is counterproductive to the strengthening of the rule of law. This is linked to the very nature of rule of law, which implies an unwritten compromise between legal and political elite regarding their role in law creation in the constitutional praxis.

## Introduction

In October 2023, in what has been termed as “the most important parliamentary election since 1989”, the three opposition alliances—“Civic Coalition” (KO), “Third Way” and “New Left”^1^ won the parliamentary majority and came to power after eight years in opposition. The national conservative, populist “United Right” composed of Law and Justice (PiS) and Sovereign Poland (SP) received the most votes, however, it had lost the coalition capacity and became the largest opposition party, next to a radical right Konfederacja.^2^ Five months later, in May 2024, the European Commission (EC) announced the end of the rule of law procedure against Poland, constating “hard work and determined reform efforts” as well as “the ongoing restoration of the rule of law in Poland”.^3^ As a result of the cooperative stance and reform proposals submitted by the new Minister of Justice, Adam Bodnar, the EC released the covid funds for Poland. However, the new government has not been able to enact any substantial measures that could heal the judiciary, primarily due to the potential veto of the PiS backed President Anrzej Duda.

Poland’s political and societal developments in the recent decade have attracted a lot of scholarly attention. Under the PiS-led government (2015–2023), the country—together with Hungary—was considered a paradigmatic case of democratic backsliding (Bakke and Sitter 2022; Sadurski 2018), autocratization and emergent illiberal regime (Pirro and Stanley 2022; Buštíková and Guasti 2017). All these concepts attest a political change and denote the gradual “stripping away of constitutional safeguards and piecemeal dismantling of democratic institutions by democratically elected incumbents” (Cianetti and Hanley 2021, p. 66). They agree on the basic observation of a movement away from democracy towards autocracy and on problematic changes in institutions and procedures leading to a state capture by dominant populist, usually illiberally inclined parties at power (Lust and Waldner 2018). In Poland, the contested “judicial reform”^4^ has triggered off particularly vehement criticism of the domestic opposition, the bulk part of judicial elite and the European Union. Most scholars have stressed the gradual subordination of the judiciary to the executive (Sadurski 2018), and the degradation of the Constitutional Tribunal to the role of “government aide” (Bugarič 2019; Castillo-Ortiz 2019), allowing to secure favorable judicial rulings. Some studies point to the opposition of civil society, particularly judicial organizations, and their “Europeanization strategies” (Grabowska-Moroz and Śniadach 2021; Moroska-Bonkiewicz and Domagała 2023), as well as to the opposition of the judges (Puleo and Coman 2024). While explaining the easiness of the “legal revolution” in some Central and Eastern European countries, scholars have paid attention to the weakness of liberal institutions and the weak institutionalization of their norms among elites and societies (Dawson and Hanley 2016).

Notwithstanding, the ruling PiS did not manage to capture judiciary to the extent as to prevent opposition parties from coming to power. I submit that Poland showcases a rule of law crisis, which can, but does not have to, lead to the establishment of an authoritarian regime. The direction of political change depends on the political will of the main actors involved in the polarization game. Whereas PiS-led government is responsible for devastation of the judiciary undermining the rule of law in the country, the new government’s continued polarizing strategy is counterproductive to the strengthening of the rule of law. This is linked to the very nature of rule of law, which implies an unwritten compromise between judicial and political elite regarding their role in law creation in the constitutional praxis. Hence, the article puts forward a theoretical argument that factors polarizing strategy of incumbents and opposition into the analysis of the rule of law crisis. Bringing together the study on pernicious polarization and party politics, I identify polarizing strategy as an explanatory factor for the observed ongoing rule of law crisis in Poland. “Polarizing strategy”—understood as an instrument of political struggle to sharpen political party identities, by reducing politics to “either-or choices” (McCoy and Somer 2021, p. 6)—has been recognized as having a detrimental effect on the quality of democracy (Somer et al. 2021; Casal Bértoa and Rama 2021; Helms 2017). This paper takes the analysis a step further and shows how severe polarization affects democracy’s central pillar, the rule of law. It also demonstrates why and how the polarizing strategy continues to have a negative effect on the rule of law even when change in power occurs. The example of Poland in the context of judicial reform acts as illustration of this process.

The article makes two main contributions to the debate. First, existing studies tend to focus on specific empirical findings. Instead, this article offers an overarching approach to studying the rule of law crisis from the perspective of agency-led polarization and provides conceptual underpinning to the debate on rule of law and polarizing politics. Second, by highlighting the role of liberal forces and their counter-polarizing strategy—first in the opposition and then in government—the paper adds an important dimension to the recognized pernicious effect of polarizing strategy on democracy and feeds into literature on democracy resilience and responsibility of political elite.

The article starts with the presentation of a theoretical argument linking the rule of law crisis and polarizing strategy. Highlighting two “radicalization mechanisms” inherent to the polarization, it shows how polarizing strategy widens the political conflict and hence touches upon the core of the rule of law. The paper proceeds with the depiction of the main tenets of the judicial reform in Poland and how it has undermined the basic principles of the rule of law in the country. It draws attention to the core of the problem: the skewed balance between “the political” and “the judicial”. It also addresses the polarizing discourse accompanying this reform on the side of the government and opposition. Finally, the paper addresses the initiatives of the new government (since December 2023) and why they have not improved the standards of the rule of law yet. The analysis highlights the possible pitfalls and challenges of the process of rule of law restoration.

## Theoretical argument: rule of law and polarization

Liberal democracy denotes the procedural consensus among elites to process policy disagreements (Sartori 1987, p. 91). Its main pillar—rule of law—does not only enables the controlled exercise of power but also protects the pluralism, and hence, the autonomy of political and societal actors in a polity. The rule of law requires political elites to agree that their power is limited and controlled. The most common definition of rule of law implies that government officials and citizens are bound by and act consistently with the law (see Tamanaha 2007). However, in a broader sense, rule of law is based on an unwritten compromise. Whereas political elite determines the direction of public policy, judicial elite maintains the right to interpret the law while applying it to respective cases. In such a way, it “co-creates” the law too. However, through their interpretation the judges (at least partially) refrain from hindering political goals inherent to the policy bills prepared by politicians (Sokołowski 2020). This “virtuous tension” between *jurisdictio* and *gubernaculum*, justice and sovereignty (Palombella 2014, 2012) renders the law autonomous from the political will. To echo Giovanni Sartori, in the case of rule of law the state is subordinated to “a law which is not its own” (see Palombella 2014, p. 6). The rule of law crisis occurs when the balance between jurisdictio and gubernaculum becomes tilted towards “the political”, and “the judicial” becomes subject of the political conflict.

Rule of law fulfils several functions that enable the realization of the central democratic principle, namely political equality. The first one, *legality*, means that the state action must comply with the law and the law defines the relationship between international and national law. *Legal certainty* implies accessibility of the law which is easy to understand and foreseeable. *Prevention of abuse of powers* includes safeguards against arbitrariness, whereas the discretionary power of the officials is limited and regulated by law. *Equality before the law* provides that the law guarantees the absence of any discrimination on grounds such as race, sex, religion, opinion etc. *Access to justice* implicates independent and impartial judiciary and the right to have a fair trial.^5^ One of the crucial indicators of fairness and impartiality of the judiciary is citizens’ trust in justice. Apart from creating the binding rules through legal decisions and regulation of societal conflict, the impartial judiciary legitimizes the state, especially among those who support opposition (Sokołowski 2017).

The ultimate institutionalization of an elite consensus leads to the establishment of a Constitutional Court, composed of judges selected by politicians and enjoying the trust of political elite. To achieve such a balanced census with the judicial elite, the political elite (in power and in the opposition) must demonstrate a minimal agreement regarding the institutional arrangement of separation of powers. A fundamental judiciary reform, which implies abandoning the hitherto valid constitutional consensus, cannot therefore succeed without a minimal consensus among political and judicial elites. Otherwise, it leads to the confrontation and broadens the scope of political conflict.

While the ruling party determines the contents of political conflict as well as related policies, the political opposition decides whether it positions itself on the opposite end of that conflict and act reactively—along the predetermined conflict axes. Alternatively, it tries to politicize new issues and thus displace the conflict, shift political alignments and hence trigger new allocation of power. “Since the development of cleavages is a prime instrument of power, the party which is able to make its definition of the issues prevail is likely to take over the government” (Schattschneider 1960, p. 73). The definition of the contents of the main conflict enables the ruling party to determine its nature, related language of politics. This is where polarization comes in. Ruling parties may try to shape identities and influence the voting behaviour through the expansion of the dominant conflict to other institutions, actors and issues. At the same time, the power of a party controlling this conflict expands as well (Schattschneider 1960, p. 17). I understand political polarization as an instrument of political struggle, whose goal is to create sharp political party identities and consolidate the political field into opposing camps (see McCoy and Somer 2021). It becomes harmful for democracy, when it divides the electorate into two mutually distrustful blocks (Somer et al. 2021).

As postulated by Somer et al. (2021), the game of polarization transforms both government and opposition. First, political polarization enjoins actors to see politics as a “battlefield between rival blocs, each posing an existential threat to the other” (Somer et al. 2021, p. 931). Subsequent *radicalization of mutual perception* leads to the situation in which legitimate rivals become enemies. Accordingly, political competition displays a “winner-takes-all logic”, both at the level of discourse and in the policymaking process. Parties, when in office, adopt a monopolistic attitude, characterized by the use of political patronage and the (policy-wise) discrimination of the opposition (Enyedi 2016). In response, opposition parties tend to resort to obstructive measures and “extra-parliamentary” practices: for instance, “street politics” or “parliamentary blockades”. Second, polarizing politics consolidates the most radical voices over potential bridge-builders within each camp (Somer et al. 2021, p. 931). This encourages *the radicalization of an appeal towards the core voters* and makes the incumbents escalate the conflict. Once the opposition comes to power, it has also to deliver on promises to their core electorate. This is why it tends to focus on settling accounts with the previously ruling camp. This, in turn, evokes a strong, obstructive reaction of the new opposition (former incumbents). As a result, the political conflict becomes even deeper. This spiral of radicalization is particularly pernicious in the case of a judicial reform.

In the polarized context, any judicial reform including (non-consulted) institutional and personnel changes will trigger off the question of *legality*, as this is the only “weapon” resisting judges have at their disposal. This automatically feeds into the narrative of opposition parties and determines their strategy of counter-polarization. As a result, the new (altered) institutions are considered partisan (if not illegal), as well as their subsequent rulings. The principle of *legal certainty* is hence likely to be undermined. Mutual distrust inherent to polarization questions the very intentions of the incumbents, so the suspicion that *democratic safeguards* preventing the abuse of power were taken over by purpose and serve the establishment of a new regime follows suit. This will raise doubts about the *impartiality* of (new) judges and hence whether *the equality before the law* can be guaranteed (especially of those with different opinion). While voicing these threats, the citizens’ trust in judiciary is likely to erode. In this way, the judiciary may lose its main function, namely the regulation of social conflict. When opposition parties come to power through the polarizing strategy and decide to sustain the polarization, they concentrate on complete delegitimization of the “reform”, trying to return to status quo ante. The new opposition will defend the reform while escalating the political conflict. In this way, the core of the problem—the skewed balance between “the political” and “the judicial” cannot be addressed, as the arguments are centered only on “whose a given institution is to be” and not on “how it should function”.

## Research design

I asses the theoretical argument by applying it to the case of Poland. The case-selection is theory-driven and proposes a crucial case to conform to the theory (Blatter and Blume 2008, p. 346). The goal is to link empirical observations back to more abstract theoretical concepts to assess their relative explanatory power. The objective is therefore not statistical generalisation towards a wider scope of cases, but analytic generalisation towards a broader theory (Yin 2009, p. 43). The examination of the rule of law crisis from the polarization perspective in Poland is highly relevant as a case study due to several reasons. In theoretical terms, Poland is the most likely case for the top-down and long-term polarization (Tworzecki 2019, Wesołowski 1997) Since 2005 the polarized competition takes place among two dominant parties Law and Justice and Civic Platform (now Civic Coalition). Both parties stem from Solidarity movement. Once the post-communist division faded, both parties needed to shape their own distinct identities, as their potential electorates were partially overlapping (Markowski 2006). This is why in the electoral campaign in 2005 PiS formulated the division between liberal and solidarity Poland. The conflict between both formations intensified during the cohabitation of the PM Tusk with the President Lech Kaczyński (2007–2010) due to the different conceptions of foreign policy and institutional-personal conflict over the role of president in shaping foreign policy. It is the Smoleńsk catastrophe in 2010 that consolidated both camps and led to the creation of a strong sense of community among PiS voters. For them the Smoleńsk investigation became a symbol of the failure of the Polish state under the Tusk government (Sokołowski 2015, p. 121–124). The contest between PiS and PO has since become a matter of Poland’s sovereignty. Both sides have seen each other as not legitimate to participate in the political competition. Moreover, the electorates of both parties differ quite considerably in terms of party loyalty, which has implications for mobilizing effect of their polarizing strategy. The core of PiS electorate is loyal and strongly identifies with the party. Around 81% of PiS voters completely trust the party—it is by far the largest group when compared to other mainstream parties. Among PO voters, only 46% declare full trust towards their party. Only 35% state that they do not have a 2nd choice party, whereas 71% of PiS voters declares so. The whole electorate of PO is linked by its anti-PiS attitude but the pro-PO arguments are very scarce. Whereas large part of PiS electorate supports party for the social transfers (Sadura and Sierakowski 2019, p. 6–33).

Poland presents also the most obvious example of rule of law crisis, as it has experienced the so-called judicial reform, embracing institutional and, above all, personnel changes. This reform has led to strong divisions among judicial elite. Poland has also experienced change in power, making it a promising context in which to study the impact of polarizing strategies on the rule of law under two different governments: The one that attempted to redesign the relation between judicial and political elites without searching for any consensus, and the one whose main declared goal is to “restore the rule of law”. In practical terms, explaining the impact of political polarization on the rule of law can yield relevant policy insights when it comes to EU attempts to counteract the rule of law crisis in the member states or to influence the rule of law reforms in the candidate states.

The empirical analysis is based on original documents (speeches, media interviews) as well as further secondary literature.

## The “judicial reform” under the PiS-led government

The functioning of judicial system in Poland before the notorious reform was frequently criticized. Long procedures, corporative character of judiciary, detachment from society, considerable influence of e.g., Supreme Court on key public policies such as privatization or lustration were among central critical points raised by experts (see e.g., Czarnota 2019). Law and Justice, in turn argued generally, that the judicial elite was out of touch with ordinary citizens and operated as a “state within a state” incapable of reforming itself (see Kaczyński 2011). According to PiS, the existing legal structure inhibited the effectiveness of the state and decisive political action. Procedures underlying the system of separation of powers between the parliament, government and judiciary were designed to serve the interests of the post-communist elite (both political and business), which, in turn, weakened the state. Kaczynski called this phenomenon “impossibilism” (see Kaczyński 2011, p. 79, the notion was often used in reference to the Constitutional Court).

The conflict around the Constitutional Tribunal (CT) in 2015 was triggered off by the previous coalition’s hasty nominations. While anticipating electoral defeat, the government’s parliamentary majority under Civic Platform (PO) and Polish People’s Party (PSL) selected five new CT judges to replace those, whose terms in office were to expire in November and December 2015, after the new parliamentary election. The CT declared, however, that the election “in advance” of only three out of five judges was constitutional. Nonetheless, the newly formed PiS government delayed the constitutionally prescribed publication of this Tribunal’s decision, which would have given it a binding force, and the President refused to swear those 3 judges into office. Instead, the new PiS majority chose five new CT judges, who were immediately sworn in by the President. According to opposition, those three “double judges” were appointed in an unlawful manner, as was the new President of the Constitutional Tribunal, Julia Przyłębska, who took up the position without the required resolution of the General Assembly of Judges of the Constitutional Tribunal. She was additionally accused by other judges of manipulating the composition of judges’ quorum while issuing different judgements.

The very judicial reform introduced by the “United Right” (PiS and Sovereign Poland of Justice Minister Ziobro) started in December 2017 and was enacted in three stages. It firstly amended the law on the National Council of the Judiciary (KRS)^6^ and changed the way this body was elected. Besides such KRS members as the President of the Supreme Court, Minister of Justice, President of the Supreme Administrative Court, a representative of the President, 4 deputies and 2 senators, the remaining 15 member-judges were appointed by the Sejm with a qualified majority of 3/5 of deputies. Should this compromise not be achieved, a simple majority sufficed. This solution implied that any ruling parliamentary majority might have a decisive influence on the composition of the KRS. The term of office of the old KRS was shortened. The second step of the judicial reform embraced Amendments to the Law on the Organisation of Common Courts. The Justice Minister received prerogatives to replace the heads of all common courts^7^ within six months. Thereafter he retained the discretionary power to dismiss court presidents for “persistent failure to comply with official duties or particularly low efficiency” (Świętochowska 2017). Third, the reform embraced also Supreme Court and provided that the retirement age of the current Supreme Court judges was lowered.^8^ PiS initially insisted that the new provision referred also to the First President of the Supreme Court^9^, Małgorzata Gersdorf, but the ruling party had to withdraw from this provision following an unfavorable ruling by the European Court of Justice (Pirro and Stanley 2022). With the new law, the President had the discretionary power to maintain some of Supreme Court judges in office, in case they submitted an adequate request within the prescribed deadline.^10^ The vacancies were to be filled with nominees selected by the newly constituted KRS. Moreover, two new chambers were introduced to the Supreme Court—a “Disciplinary Chamber” and a “Chamber of Extraordinary Control and Public Affairs”. The Disciplinary Chamber had the power to decide on disciplinary charges against all judges in the country and its members received higher renumeration than other judges of the Supreme Court. The Extraordinary Control and Public Affairs Chamber was to decide on validity of elections as well as on the possibility to repeal final decisions of courts in a newly created extraordinary appeal procedure. The members of both new chambers were to be elected by the new KRS.

Indeed, most controversies were linked to the way the reform was introduced and the new posts were filled. As opposition parties regarded the amendments to the law on KRS as unconstitutional, they boycotted the election of new judges by Sejm. As a result, the new KRS was composed of candidates supported by PiS, Sovereign Poland, and Kukiz’15.^11^ The list of candidates was very short (18 for 15 places) as many judges also boycotted the whole election process. Thus, 12 selected judges were linked to the General Prosecutor (Ministry of Justice), and further members of the KRS were former deputies of PiS (Sokołowski 2020). Since then, the opposition politicians and their sympathizers have called the body “neo-KRS” and judges elected by that body—“neo-judges”, while questioning the legality of their status as judges, and hence of their rulings. Similarly, the bulk part of judges boycotted the selection process to the disciplinary chamber. As a result, only 10 (out of 16) were finally appointed (Sokołowski 2020). All of them had close links either to the General Prosecutor (Minister of Justice) or to PiS.

In December 2019, the Polish Supreme Court ruled that in its current composition, the new KRS was neither impartial nor politically independent. Additionally, the disciplinary chamber was not a court in the light of EU law and hence it was not a court in the light of national law.^12^ In January 2020 three chambers of the Supreme Court issued a resolution stating that the court formations including Supreme Court judges appointed through the procedure involving the new KRS were unduly composed.^13^

In response, PiS-led government introduced new disciplinary measures, including fines, forced movement to other courts or removal from office. They were to be used against judges for actions that impede the functioning of the judicial system, question the legitimacy of other judges’ appointment and for public activities that are incompatible with the principles of judicial independence.^14^ The PiS-led government justified these measures by arguing that questioning the legitimacy of new judges appointed by President would destabilize the whole judicial system (Świętochowska 2017). The disciplinary measures, which opposition dubbed a “muzzle law” (“ustawa kagańcowa”), could further undermine judicial independence by serving to intimidate critical judges.^15^ In sum, the way the posts in new KRS and within Supreme Court were filled bore the threat of politicization of both bodies, and their dependence on Minister of Justice/General Prosecutor, hence—on executive.

## Discussion: polarizing strategy and the rule of law crisis

Because the PiS-led government conducted the judicial reform without searching for any consensus among political nor judicial elites, the result of such policy is a delegitimization of the new KRS and the Supreme Court within the sphere of judicial elites, which has frequently been visible in internal conflicts and opposing resolutions. Hence, the first principle, *legality*, has been violated by the fact that some judges question the lawfulness of the new KRS and the judges appointed by it. Subsequently the principle of *legal certainty* is undermined as the judgments issued by these judges are also being questioned. In fact, between 2018 till 2023 nearly 2205 judges were nominated by the new KRS^16^ and all their rulings could potentially be challenged. The way the judges’ selection has occurred threatens their independence and impartiality. The way the Constitutional Tribunal has become subordinated to the ruling majority points to the violation of one of the democratic safeguards against the arbitrary political decisions. In praxis it means that parliamentary opposition parties have lost one of their control instruments, namely the effective possibility of judicial review.

The position of both camps has quickly become irreconcilable, while they tried to mobilize their own voters in the runup to 2019 and then 2023 parliamentary election. Opposition and most of judicial organizations claimed that the basic standards of judicial independence and impartiality were violated, Poland stopped being a constitutional state based on the rule of law and the judiciary was subordinated to the executive. This threatened Poland’s EU membership and the protection of human rights and freedoms, as the judicial rulings could be instrumentalized to the benefit of the incumbents. This, in turn could pave the way to the “Polexit”. The latter argument was particularly audible when the Polish Constitutional Tribunal ruled that EU institutions applied some provisions of the main EU treaty in a way that went beyond their legal competencies transferred to them by Poland. Additionally, opposition claimed that PiS would not accept the electoral defeat and would do anything to stay at power.

PiS, in turn, argued that the introduced changes did not violate the rule of law and were necessary because the judges were corrupt and constituted a “caste”, who issued unjust rulings and hence deteriorated the life of many people. These claims were accompanied by the aggressive “fair courts” campaign against judges in state TV and on the billboards.^17^ Further claims concerned the accusations that judges themselves did not want to observe the Constitution and while trying to protect their privileges referred to the EU Court of Justice and the precedence of the EU law over the national law.

The core of the problem appears, however, in breaking the hitherto valid, unwritten consensus among political and judicial elites as to the systemic position of judicative and its role in law creation, or what Palombella (2014, p. 6) called “the second pillar of the law”. This consensus has been abandoned by the PiS government in the way that the balance between *jurisdictio* and *gubernaculum* has been clearly tilted towards “the political”, which undermines the rule of law. In the constitutional praxis it may lead to the rule *by* law, i.e. where the legislator, the parliamentary majority, owns (creates and interprets) the law it enacts without a judicial control. In order to restore the meaningful rule of law, a new compromise between judicial and political elite on the one hand, and a minimal agreement between opposition and ruling elite on the other, are necessary. The continued polarization in Poland renders such compromise impossible.

## “Restoration of the rule of law” under the new ruling coalition

According to the new coalition’s treaty, its main task is to “restore the rule of law” and “repair the state”.^18^ However, the potential veto of the PiS-backed President on the one hand, and the lack of programmatic cohesion in both socioeconomic and cultural issues on the other hinder the advancement of related policies. The new government is concentrating primarily on the measures including reckoning (rozliczenia) with the previous PiS-led government over its abuse of power. The instruments for this strategy are e.g. investigative commissions. In terms of policy changes, it is prioritizing two key measures: elite replacement and reversal of most contested policies introduced by previous incumbents. The former appears unproblematic as far as appointments that need a simple resolution are concerned, such as those in public administration, government supervisory bodies, state companies. But in key institutions, such as Constitutional Tribunal, the Prosecution Office and Supreme Court, PiS has entrenched its political influence through the nominees, whose terms in office are either constitutionally protected or their ending depends on the President. The new government has been trying to overcome these constraints by passing parliamentary resolutions that question some of Law and Justice’s appointments (invoking rulings by the EU courts), or ministerial directives that are not subject to presidential veto nor a referral to Constitutional Tribunal. Both measures have been encountered by widespread criticism of constitutional experts and international organizations. Even though the damaging judicial reform has been introduced on the basis of the parliamentary bills—what Kim Scheppele (2018) has termed “autocratic legalism”—the restoration of the rule of law needs also to proceed within legal framework. But the crucial point is, that the perception of what legal is, differs among polarizing actors.

The apparently insurmountable conflict over judiciary has become most visible within the axis President—government. Whereas the President has not agreed to question the status of judges nominated by the new KRS to the Supreme Court, Constitutional Court or common courts, sworn in so far, for the government, the elimination of the judges nominated by the new KRS appears as a primary condition for further reforms. In the first step, the parliament introduced a resolution on *Constitutional crisis and the Constitutional* Tribunal stating that the CT is not fulfilling its functions and hence, is de facto non-existing.^19^ This move was unprecedented and did not have any constitutional base. Since then, the government has not been publishing the CT judgements, which is constitutionally prescribed. At the same time, common courts do apply the rulings of CT issued after 2017. So far only one policy proposal has been presented to restore independence of the Constitutional Tribunal—a proposal based on civil project drafted by Stefan Batory Foundation.^20^ It suggests, among others, the selection of CT judges through 3/5 of votes in Sejm. However, the solution is unlikely to be implemented within the polarized scene.

In the same vein, the ruling coalition considers the current KRS as de facto non-existing, which was confirmed in the government’s resolution on “Counteracting the negative consequences of the constitutional crisis within judiciary”^21^ in December 2024. In terms of constituting a new, lawful KRS, the ruling majority has approved a bill whereby members of the KRS are to be chosen in secret and direct elections held among judges themselves. In terms of passive right to candidate to the future KRS, the Senate’s amendment providing that the judges nominated after 2018 by the current KRS could candidate again to the future KRS, was rejected by Parliament upon the recommendation of Justice Minister.^22^ At the same time, however, in 2024, the ruling parliamentary majority chose four new members to the (still unreformed) KRS and in this way legitimized this organ.

The most pressing problem is the status of judges appointed by the KRS after 2018. In September 2024, Justice Minister presented further proposals: some of the judges would be forced to return to the previous jobs, others, who graduated from Polish School of Judiciary and Prosecution would be able to maintain their status of judges. Moreover, the draft implied the liquidation of two new chambers within the Supreme Court, whose status has been questioned by the EUCJ. In general, the Venice Commission critically assessed the differential treatment of the judges appointed after 2018 and highlighted their right to appeal.^23^ It further argued that their potential impartiality stemmed primarily from wrong procedures and the individualized assessment of how they fulfilled their duties was needed.

Finally, the government ignores also the rulings of the Supreme Court, especially those issued by the two contested chambers. In January 2024, the Minister of Justice removed the National Prosecutor, Dariusz Barski, from his office on the basis of legal expertise stipulating that his nomination was based on law that was not valid at that time.^24^ However, the removal of National Prosecutor from office requires the acceptance of the President. What is more, in September 2024 the Disciplinary Chamber of the Supreme Court issued a resolution stating that the nomination of Barski was legal and binding.

## Discussion: polarizing strategy and the rule of law crisis

Whereas the principle of legality might be formally restored if the KO candidate wins presidential election in May 2025 and the new related bills will be signed into law, the perception of politicized institutions among current opposition and its constituency will not disappear, neither the fear of dependent judges or unfair trials. The rule of law standards have not improved in the first year of the new government. The most illustrative example is the arbitrary withdrawing of counter-signature by PM Tusk, who, by mistake, granted his counter-signature to the President’s appointment of a judge (nominated by the current KRS) to the Supreme Court.^25^ Polish law does not provide for the withdrawal of counter-signature, once it has been granted by the Prime Minister.

The new coalition has regarded the key institutions established or changed by the PiS-led majority: KRS, Supreme Court and Constitutional Tribunal as non-constitutional and hence illegal. Despite this perception, these institutions work and issue judgements, which results in inconsistent treatment of their rulings by the government. Some constitutionally questionable moves, e.g. the removal of National Prosecutor, have fueled the accusation of deepening of rule of law crisis.^26^

In September 2024 Prime Minister Tusk stated that the government was forced to act in the mode of “militant democracy”.^27^ In this way, he tried to justify some of legally more questionable moves, on the grounds that constitutional safeguards can sometimes be ignored to restore legal order to institutions that have been corrupted or whose legitimacy is questionable. Nonetheless, the pure delegitimization of institutions that were taken over by PiS has not resulted in strengthening of the rule of law. This is the logic of polarization which traps the government in its monopolistic attitude and confrontational style and then the opposition (which may become the new government) in the *reactive* mode—which hinders a more positive and credible forward-looking policy agenda including resolving some real, structural problems of the judiciary. It also hinders a necessary compromise on the scope of judicial influence on public policy.

## Conclusion

Polarizing strategy contributes to rule of law crisis in several ways. Polarizing actors introduce policy changes in a one-sided manner which leads to confrontational reaction of opposition. The mutual trust erodes, the affected institutions become politicized in the eyes of opposing actors. While escalating the conflict in the political discourse they also radicalize the appeal toward voters and adjust their strategies accordingly. For opposition it means mobilizing voters on the anti-incumbent stance. After the change in power this anti-incumbent focus determines the governing style and priorities—more reckoning, and policy reversals, but less pro-active initiatives addressing the real problems.

The problem of the rule of law in Poland is two-fold. First, the judicial reform accompanied by polarizing politics of the PiS-led government has led not only to the confrontation with the domestic opposition and the EU establishment, but also to the conflict within the judicial elite themselves. This has influenced the functioning and legitimacy of the key institutions in the country and increasingly affects the life of citizens.^28^ Second, even though the counter-polarizing strategy of the main opposition alliance KO (since December 2023 the leading party in the government) may have been conducive to uniting and mobilizing voters in the runup to the parliamentary election in October 2023, the continuation of this strategy, while in government, hinders the necessary political reforms that could depoliticize key institutions like Constitutional Tribunal or National Judiciary Council. It hence contributes to the political conflict over the judiciary. This paper confirms that the strategies the political actors choose, go beyond their usual performance (Ilonszki et al. 2021). They have much broader, system-related implications, especially when it comes to the establishment of a balanced separation of powers.

Whereas the polarizing strategy might have been useful for the KO in government during the recent sequence of elections (regional election, election to EU Parliament in 2024 and presidential election in May 2025), allowing it to mobilize people on the same anti-PiS appeal, in the long-term perspective, the more critical and less attached voters might become disillusioned about the ongoing Polish-Polish war and lack of reform progress. Given the loyalty of the PiS electorate, the polarizing strategy appears more beneficial for them. However, polarizing strategy has broader effects on democratic institutions. Polarized blocks of supporters become impervious to internal criticism and this hinders any compromise, but also weakens the control and accountability mechanisms within society. This was one of the arguments voiced by Svolik (2020) that polarization fosters illiberal governments backed by voters. In a polarized context, elections become instruments for plebiscitarian legitimation rather than an instrument to elect representatives to lead and to make decisions for which they later will be made accountable. Sustaining democratic order—like a successful democratization process, where the judiciary was still in the making—requires elites’ self-restraint when drafting a new elites’ compromise. The sole party interests cannot dictate the terms of such compromise.

## Footnotes

1 The centrist-liberal “Civic Coalition” is an electoral alliance, composed of “Civic Platform” (PO), the “Modern” (.N), “Polish Initiative” and “the Greens”, they constitute one parliamentary faction in the current legislature. The “Third Way” includes the moderate conservative “Polish People’s Party” (PSL) and Poland 2050 of Szymon Hołownia, both have created two separate parliamentary factions; the “New Left” is a merger between the Democratic Left Alliance (SLD) and the “Spring”. The party has built a parliamentary faction named “the Left” with the “Labour Union”, “Together” and “Polish Socialist Party” (PPS).

2 PiS won the largest share of the vote with 35.4%, but this only translated into 194 seats in the 460-member Sejm, well short of the 231 required for an overall majority.

3 https://ec.europa.eu/commission/presscorner/detail/hu/ip_24_2461.

4 There is a consensus among scholars that the reform has boiled down primarily to the change of elite in the main state institutions but the key mechanisms that hinder e.g. the efficiency of courts have not been reformed. The analysis of the “judicial reform” in this paper includes Constitutional Tribunal, National Judiciary Chamber (KRS) and the Supreme Court.

5 The Venice Commission proposes these five core elements of the rule of law to assess the level of rule of law compliance in a given state: https://www.venice.coe.int/WebForms/pages/?p=02_Rule_of_law&lang=EN.

6 The KRS assesses and proposes the candidates for judges of the Supreme Court as swell as ordinary, administrative and martial courts.

7 The Justice Minister replaced 130 presidents and vice-presidents of the courts (out of 730) within the granted 6 months, https://www.rp.pl/Sedziowie-i-sady/302139943-Minister-Sprawiedliwosci-Zbigniew-Ziobro-wymienia-prezesow-12-sadow.html.

8 *The “Supreme Court judges shall not be removable” (Art. 180, Constitution 1997).*

9 The Constitution of 1997 clearly states that “the First President of the Supreme Court shall be appointed by the President of the Republic for a 6-year term of office…” (Art. 183.3.).

10 The President has maintained some judges and dismissed others without justification, even if they had submitted their request for the tenure prolongation in accordance with the new law. For more details see https://www.rp.pl/Rzecz-o-prawie/310069996-Sadownictwo-na-rowni-pochylej-sprawy-zaszly-za-daleko---Mariusz-Krolikowski-o-wojnie-sadowej.html.

11 Kukiz’15 was an opposition party, which, in contrast to remaining parliamentary factions on the side of opposition, cooperated with the PiS-led government. On the basis of an agreement it supported some of PiS policies and—in return—PiS enacted some of Kukiz’15 key policy proposals.

12 https://www.sn.pl/aktualnosci/SitePages/Komunikaty_o_sprawach.aspx?ItemSID=331-b6b3e804-2752-4c7d-bcb4-7586782a1315&ListName=Komunikaty_o_sprawach.

13 https://www.sn.pl/aktualnosci/SitePages/wydarzenia.aspx?ItemSID=598-0dc69815-3ade-42fa-bbb8-549c3c6969c5&ListName=Wydarzenia.

14 https://prawo.gazetaprawna.pl/artykuly/1454011,ustawa-kagancowa-kiedy-wejdzie-w-zycie.html.

15 Even though the government’s opponents had argued continually (both on domestic political scene and within the EU Parliament) that the “muzzle law” would further undermine the rule of law in Poland, many opposition deputies did not appear to the voting over the first reading of that bill. Many PiS deputies were also not present in the Sejm, so the opposition had a real chance to reject the bill https://www.rp.pl/polityka/art974341-poslowie-opozycji-tlumacza-sie-z-nieobecnosci-na-glosowaniu.

16 https://hfhr.pl/upload/2023/10/raport_powolania_sedziow_przez_nowa_krs.pdf.

17 The “fair courts” campaign conducted by the Polish National Foundation implied renting billboards to present cases where courts and judges had made allegedly wrong decisions or committed crimes. Most information on those billboards was, however. selective and misinterpreted.

18 https://www.gov.pl/web/premier/koalicja-15padziernika-program.

19 https://orka.sejm.gov.pl/proc10.nsf/uchwaly/226_u.htm.

20 https://www.batory.org.pl/2024/03/04/spoleczny-projekt-ustaw-o-trybunale-konstytucyjnym-skierowany-do-prac-parlamentarnych/.

21 https://www.gov.pl/web/premier/uchwala-w-sprawie-przeciwdzialania-negatywnym-skutkom-kryzysu-konstytucyjnego-w-obszarze-sadownictwa.

22 https://www.pap.pl/aktualnosci/bodnar-nie-wycofalem-sie-z-poparcia-senackiej-poprawki-dot-krs-wywiad.

23 https://www.venice.coe.int/webforms/documents/default.aspx?pdffile=CDL-PI(2024)009-e.

24 https://www.gov.pl/web/sprawiedliwosc/informacja-o-uznaniu-przywrocenia-prokuratora-dariusza-barskiego-do-sluzby-czynnej-za-akt-dokonany-z-naruszeniem-przepisow.

25 https://www.pap.pl/aktualnosci/eksperci-o-uchyleniu-kontrasygnaty-przez-premiera-tuska-komentarze.

26 Most recently, the President of the Constitutional Tribunal accused PM Tusk to lead an “organized criminal group” aiming to change the political order while disempowering Constitutional Tribunal, Supreme Court and the KRS, see https://trybunal.gov.pl/wiadomosci/uroczystosci-spotkania-wyklady/art/x.

27 https://www.gov.pl/web/premier/dzialamy-w-imie-demokracji-walczacej.

28 Only in the Supreme Court, in the years 2022–2024, contested judgments in 155 criminal cases were overturned because they were issued by the co called neo-judges. https://www.sn.pl/aktualnosci/SitePages/Wydarzenia.aspx?ItemSID=1050-0dc69815-3ade-42fa-bbb8-549c3c6969c5&ListName=Wydarzenia
